# Growth promotory potential of the cold adapted diazotroph *Pseudomonas migulae* S10724 against native green gram (*Vigna radiata* (L.) Wilczek)

**DOI:** 10.1007/s13205-014-0259-0

**Published:** 2014-10-15

**Authors:** Deep Chandra Suyal, Anjana Shukla, Reeta Goel

**Affiliations:** Department of Microbiology, College of Basic Sciences and Humanities, G.B. Pant University of Agriculture and Technology, Pantnagar, 263145 Uttarakhand India

**Keywords:** Diazotroph, Himalayan belt, Bioinoculant, Plant growth promotion

## Abstract

It is being confirmed previously the atmospheric nitrogen fixing ability of the cold adapted *Pseudomonas migulae* S10724 strain at the fluctuating temperatures. Therefore, net house bioinoculation experiment was performed to determine the effectiveness of inoculation of strain S10724 on the growth enhancement of native green gram (*Vigna radiata* L. Wilczek). The strain significantly (*p* < 0.05) stimulated the growth of roots (45.3 %) and shoots (45.6 %) of green gram plants. Furthermore, other growth related parameters viz. fresh and dry weight was also found to be increased significantly. Treated plants typically showed more obvious modifications in their biochemical status also. The total chlorophyll and nitrate reductase activity was increased in S10724 inoculated plant as compared to the control one. Moreover, in vitro seed germination assay revealed that the germination was increased in S10724 strain treated seeds by 22 % at 25 °C while 25 % at 12 °C unlikely to respective controls. The results suggest that *P.migulae* S10724 strain is a potential plant growth promoting bacterium for legume under fluctuating temperature ranges and therefore, could be used effectively as a low cost bioinoculant in Himalayan agricultural belt successfully.

## Introduction

The Himalaya has the epithet of being one of Earth’s most diverse ecosystems—justified by the largely unparalleled variation in topography, vegetation patterns, climate and inhabitants. Extreme climatic conditions because of the diurnal fluctuations in temperature, scanty rainfall, low air pressure and strong solar radiation play a critical role in the selection of the efficient microorganisms. The low productivity of cereals, oilseeds, pulses in such fragile ecosystems with treacherous mountainous terrain is a major obstacle for obtaining the sustainable agricultural productivity.

The productivity of pulses is very poor in upper Himalayas. They are grown as mixed with major cereals and minor millet crops during kharif as a measure to ensure the food security (Mehta et al. 2010). Green gram, a legume likely native to India, is widely grown and consumed as sprouts or dry beans. Green grams are generally warm-season, deep-rooted plants whose specific hardiness and day-length requirements vary by cultivar, though most require from 90 to 120 frost-free days annually (Ranawake et al. 2011). These conditions are very hard to achieve in the high altitude agriculture lands and therefore, their production in the Himalayan hilly regions is almost negligible. However, development of polyphasic agricultural strategies viz. reshaping the rhizosphere microbiome in favor of microorganisms that improve plant productivity and prevent the crop plants against the biotic and abiotic stresses could be an effective environmental friendly alternative. It has been postulated that the rhizospheric microorganisms contributes to the ability of some plant species to survive under extreme conditions (Jorquera et al. 2012). Nevertheless, it is interesting to note that despite the impact of low temperatures on nodule formation and nitrogen fixation, native legumes in the high arctic can nodulate and fix nitrogen at rates comparable to those reported for legumes in temperate climates (Bordeleau and Prevost 1994). Therefore, there is great interest in agriculture for microbial bio-inoculants that enhance growth of plants under cold conditions.

*P. migulae* S10724 (JX173286) was originally isolated from the rhizosphere of red kidney bean (*Phaseolus vulgaris* L.) from Chhiplakot (3,290 m, 30.06°N, 79.01°E), a Western Indian Himalayan high alpine meadows (Suyal et al. 2014). Differential proteomic analysis of bacterium S10724 revealed that under the low temperature diazotrophy, most of the upregulated proteins was stress proteins, while majority of the downregulated proteins were related to cell division (Suyal et al. 2014). This was the first major effort of the isolation and differential proteomic analysis of cold adapted diazotroph from Himalayan high altitude rhizospheric soil, while, present study subsequently explore its growth promontory potential under natural fluctuating temperatures.

## Materials and methods

The pot trial was performed at Pantnagar (244 m, 28.97°N, 79.41°E), a *Tarai* region of Indian Shiwalik Himalayas, during the month of Oct to Nov. In brief, the soil was loam in texture and soil pH was 7.2. Pots were kept in a net house having natural fluctuating temperature range from 30 ± 5 °C in day to 10 ± 5 °C in night, for 60 days (Katiyar and Goel 2003; Rani et al. 2012). The psychrophilic diazotroph *P. migulae* S10724 was cultivated aerobically in Burk medium at 12 °C (Suyal et al. 2014). The seeds of green gram were bacterized (10^8^ cells/seed) using carboxymethyl cellulose with strain S10724 (Katiyar and Goel 2003). Nonbacterized seeds served as a control. Three replicates for each treatment were taken. Agronomical parameters of plants were measured after harvesting. The chlorophyll content of the plant leaves was measured using the method described by Tripathy et al. (2007); while, the nitrate reductase activity of plant flag leaves was measured according to Hageman and Hicklesley (1971).

In vitro comparative seed germination assay was also conducted to determine the effect of Strain S10724 on the green gram seed germination under low temperature (12 °C) and ambient temperature (25 °C). In experiment, 25 green gram seeds were imbibed in 5 ml of a 1 × 10^8^ ml^−1^ bacterial suspension. Controls were imbibed with sterile distilled water only. After 15 min excess suspension was decanted off and the seeds were plated out on to filter paper laid over 0.5 % water agar in Petri dishes (10 seeds per Petri dish) at 12 and 25 °C under a diurnal cycle of white light. The number of seeds germinated was recorded as seedlings with coleoptile lengths >5 mm on day 3 and 6 post-inoculation. The number of germinating seeds was taken as the mean of three Petri dishes so that each value was the mean of 30 imbibed seeds (three Petri dishes × 10 seeds), expressed as a percentage of the controls.

## Results and discussion

Microorganisms play a pivotal role in the functioning of crops by influencing their physiology and development. Rhizosphere microorganisms promote plant growth and protect plants from pathogen attack by different mechanisms (Mendes et al. 2013). These involve biofertilization, stimulation of root growth, rhizoremediation, control of abiotic stress, and disease control. It is now well known that the rhizospheric microorganisms help the plant species to survive under extreme conditions (Jorquera et al. 2012). In this perspective, exploration of cold-adaptive bacteria as representative candidate of indigenous biodiversity could be a better alternative for improved crop protection and sustainability.

The effect of *P. migulae* S10724 strain was significantly greater with an increase in root length of 45.3 %, and shoot length of 45.6 %, respectively, over control (Table 1). The total chlorophyll content was also significantly higher in strain S10724 inoculated plants and increased by 40 %. Increased chlorophyll content and, subsequently, enhanced photosynthesis, is a known plant response to inoculation with several plant growth promoting bacteria (PGPBs) (Alam et al. 2001; Rani et al. 2012). It was confirmed that the increased production of photosynthesis enhanced plant growth and yield (Panwar 1991; Singh et al. 2012). Inoculation of green gram plants with S10724 strain increased nitrate reductase activity compared with uninoculated plants by 80.3 % (Table 1). The reduced nitrogen input to the plant is determined by the activity of nitrate reductase, which catalyzes the first step and determines the rate of this assimilating process that acts as a limiting factor of plant growth and development (Solomonson and Barber 1990). Plant biomass was also found to increase significantly by 48.8 and 67.79 % by measuring fresh and dry weights, respectively. Microorganisms play a pivotal role in the functioning of crops by influencing their physiology and development. Rhizosphere microorganisms promote plant growth and protect plants from pathogen attack by different mechanisms (Mendes et al. 2013). These involve biofertilization, stimulation of root growth, rhizoremediation, control of abiotic stress, and diseases. Moreover, it is now well known that the rhizospheric microorganisms help the plant species to survive under extreme conditions (Jorquera et al. 2012). Results of in vitro seed germination assay confirm the efficacy of S10724 strain to enhance the green gram seed germination. Differences in germination, relative to the controls were observed after imbibing seeds with bacterial suspension. The germination was increased in treated seeds by 22 % at 25 °C with 3 days germination time while 25 % at 12 °C but with longer germination period (6 days) (Table 1).

**Table 1:** *t* test analysis depicting the effect of psychrophilic diazotroph *Pseudomonas migulae* strain S10724 on mung bean under net house conditions after 60 days of germination

Treatment	In vitro seed germination assay		Pot trial data					
	% germination of the seeds							
	12 °C^a^	25 °C^a^	Shoot length (cm)	Root length (cm)^a^	Plant fresh weight (g plant^−1^)^a^	Plant dry weight (g plant^−1^)^a^	Chlorophyll content (mg g^−1^ fr. wt)^a^	Nitrate reductase activity (mmol NO_2_ g^−1^ fr. wt h^−1^)^a^
Control	30 ± 0.51 (6)^b^	70 ± 0.26 (3)^b^	7.22 ± 0.84	5.67 ± 0.65	0.44 ± 0.13	0.19 ± 0.43	0.40 ± 0.07	0.25 ± 0.16
*P. migulae* S10724	40 ± 0.32 (6)^b^	90 ± 0.12 (3)^b^	13.26 ± 0.55 (45.6)^c^	10.36 ± 1.02 (45.3)^c^	0.86 ± 0.21 (48.8)^c^	0.59 ± 0.61 (67.79)^c^	0.69 ± 0.16 (42)^c^	1.27 ± 0.16 (80.3)^c^
*f* value	3.18	5.35	1.29	7.28	7.97	5.86	5.11	1.18
*t* value	5.21	4.01	5.62	3.84	5.61	5.39	3.76	6.71

Data were analyzed statistically at the 5 % *p* (<0.05 %) level of significance

^a^Each value is mean of five replicates

^b^Values in parentheses indicate germination time in days

^**c**^Values in parentheses indicate percent increase over treatment

Colonization of soil by non-indigenous microorganisms depends on an interaction with indigenous microflora associated with plants, and also the ability to utilize diverse substrates in the soil (Miethling et al. 2000) Present study revealed that *P. migulae* S10724 strain is an efficient rhizosphere colonizer, which was clearly evident from the significant increments in plant growth parameters under an unsterilized soil system. These findings are in agreement with those of Katiyar and Goel (2003) who assessed the inoculation effect of phosphate-solubilizing cold-tolerant mutant of *P. fluorescens* on mung bean in sterilized and unsterilized soil. Katiyar and Goel (2003) observed that inoculated plants resulted in better plant growth in both soils. This implies that rhizobacteria had more competitive ability to survive and affect the growth of inoculated plants in the presence of indigenous microflora. Furthermore, Trivedi and Sa (2008) found two mutants that were more efficient than the wild-type strain *Pseudomonas corrugata* in phosphorus solubilization across a temperature range from 4 to 28 °C. There are different reports on the successful implementation of the microorganisms as the PGPBs (Kogel et al. 2006; Singh et al. 2012; Rani et al. 2012; Qiang et al. 2012). Selvakumar et al. (2013) revealed the solubilization of rock phosphate using *Pseudomonas* spp. isolated from the rhizoplane of wild grass from Indian Himalayas. Recently, Joshi et al. (2014) analyzed the diversity of cold-tolerant phosphate-solubilizing microorganisms from North Western Himalayas. Mendes et al. (2013) reviewed the significance of rhizosphere microbiome under several biotic and abiotic stresses but the exploration of the psychrophilic diazotrophs for enhancing plant growth is still lacking. It is probably due to unavailability of such strain(s). Therefore, this study is unique and can fill-up this gap.

## Conclusion

In conclusion, rhizospheric microorganisms can alleviate abiotic stresses on crops, providing an environmentally sound alternative for genetic engineering and plant breeding. However, successful implementation of microbial bio-inoculants is dependent on shelf-life, variable efficacy across environments and different plants species etc. To resolve them, more fundamental knowledge is required about their behavior and mutual interactions. To the best of our knowledge, this preliminary study for the first time provides an overview about the cold adapted diazotroph mediated plant growth promotion which will definitely facilitate the development of microbial inoculants for the agriculture in fluctuating hill environments.
